# Supplementary material for: G ATA2 mediates the negative regulation of the prepro-thyrotropin-releasing hormone gene by liganded T3 receptor β2 in the rat hypothalamic paraventricular nucleus
Source: PLoS One. 2020 Nov 17;15(11):e0242380. doi: 10.1371/journal.pone.0242380 (PMC7671546; doi:10.1371/journal.pone.0242380)
Supplement: S1 File — (PDF) [file pone.0242380.s001.pdf]

ATCC

Product Sheet

CA-77 (ATCC® CRL-3234™)

Please read this FIRST

Storage Temp.
liquid nitrogen vapor phase

Biosafety Level
1

Intended Use

This product is intended for research use only. It is not intended for any animal or human therapeutic or diagnostic use.

Complete Growth Medium

The base medium for this cell line is ATCC-formulated DMEM:F12 Medium Catalog No. 30-2006. To make the complete growth medium, add the following components to the base medium: fetal bovine serum to a final concentration of 10%.

Citation of Strain

If use of this culture results in a scientific publication, it should be cited in that manuscript in the following manner: CA-77 (ATCC® CRL-3234™)

American Type Culture Collection
PO Box 1549
Manassas, VA 20108 USA
www.atcc.org

800.638.6597 or 703.365.2700
Fax: 703.365.2750
Email: Tech@atcc.org

Or contact your local distributor

Description

Organism: *Rattus norvegicus*, rat
Tissue: thyroid C cells
Disease: medullary thyroid carcinoma
Morphology: neuronal-like
Growth Properties: adherent

Batch-Specific Information

Refer to the Certificate of Analysis for batch-specific test results.

SAFETY PRECAUTION

ATCC highly recommends that protective gloves and clothing always be used and a full face mask always be worn when handling frozen vials. It is important to note that some vials leak when submersed in liquid nitrogen and will slowly fill with liquid nitrogen. Upon thawing, the conversion of the liquid nitrogen back to its gas phase may result in the vessel exploding or blowing off its cap with dangerous force creating flying debris.

Unpacking & Storage Instructions

1. Check all containers for leakage or breakage.
2. Remove the frozen cells from the dry ice packaging and immediately place the cells at a temperature below -130°C, preferably in liquid nitrogen vapor, until ready for use.

Handling Procedure for Frozen Cells

To insure the highest level of viability, thaw the vial and initiate the culture as soon as possible upon receipt. If upon arrival, continued storage of the frozen culture is necessary, it should be stored in liquid nitrogen vapor phase and not at 70°C. Storage at 70°C will result in loss of viability.

1. Thaw the vial by gentle agitation in a 37°C water bath. To reduce the possibility of contamination, keep the O-ring and cap out of the water. Thawing should be rapid (approximately 2 minutes).
2. Remove the vial from the water bath as soon as the contents are thawed, and decontaminate by dipping in or spraying with 70% ethanol. All of the operations from this point on should be carried out under strict aseptic conditions.
3. Transfer the vial contents to a centrifuge tube containing 9.0 mL complete culture medium and spin at approximately 125 x g for 5 to 7 minutes.
4. Resuspend cell pellet with the recommended complete medium (see the specific batch information for the culture recommended dilution ratio) and dispense into a new culture flask. It is important to avoid excessive alkalinity of the medium during recovery of the cells. It is suggested that, prior to the addition of the vial contents, the culture vessel containing the complete growth medium be placed into the incubator for at least 15 minutes to allow the medium to reach its normal pH (7.0 to 7.6).
5. Incubate the culture at 37°C in a suitable incubator. A 5% CO_2 in air atmosphere is recommended if using the medium described on this product.

Subculturing Procedure

Volumes used in this protocol are for 75 cm² flasks; proportionally reduce or increase amount of dissociation medium for culture vessels of other sizes.

1. Remove and discard culture medium.
2. Briefly rinse the cell layer, twice, with 5 mL Ca++/Mg++ free Dulbecco's phosphate-buffered saline (D-PBS) or 0.25% (w/v) Trypsin - 0.53 mM EDTA solution to remove all traces of serum which contains trypsin inhibitor.
3. Add 5 mL of 0.25% Trypsin-EDTA solution to flask and observe cells under an inverted microscope until cell layer is dispersed (usually within 5 to 15 minutes).

   Note: To avoid clumping do not agitate the cells by hitting or shaking the flask while waiting for the cells to detach. Cells that are difficult to detach may be placed at 37°C to facilitate dispersal.
4. Add 5 mL of complete growth medium and aspirate cells by gently pipetting.
5. Transfer cell suspension to a centrifuge tube and spin at approximately 125 x g for 5 to 10 minutes. Discard supernatant.
6. Resuspend the cell pellet in fresh growth medium. Add appropriate aliquots of the cell suspension to new vessels. An inoculum of 7.0 X 1^4 to 1.0 X 10^5 viable cells/cm² is recommended.
7. Incubate cultures at 37°C.

Subcultivation ratio: A subcultivation ratio of 1:3 to 1:5 is recommended.

ATCC

Product Sheet

CA-77 (ATCC® CRL-3234™)

Please read this FIRST

Storage Temp.
liquid nitrogen vapor phase

Biosafety Level
1

Intended Use

This product is intended for research use only. It is not intended for any animal or human therapeutic or diagnostic use.

Complete Growth Medium

The base medium for this cell line is ATCC-formulated DMEM:F12 Medium Catalog No. 30-2006. To make the complete growth medium, add the following components to the base medium: fetal bovine serum to a final concentration of 10%.

Citation of Strain

If use of this culture results in a scientific publication, it should be cited in that manuscript in the following manner: CA-77 (ATCC® CRL-3234™)

American Type Culture Collection
PO Box 1549
Manassas, VA 20108 USA
www.atcc.org

800.638.6597 or 703.365.2700
Fax: 703.365.2750
Email: Tech@atcc.org

Or contact your local distributor

Medium renewal: Every 2 to 3 days

Cryopreservation Medium

Complete growth medium, 95% supplemented with 5% DMSO

Comments

CA77 cells have a neuronal-like phenotype, neurite extension is enhanced by growth on laminin substrate. Cells have a neural crest derived lineage, which makes them a useful model for differentiation studies; expresses calcitonin gene-related peptide (CGRP) and other neuropeptides, which along with the serotonergic neuronal-like phenotype, makes them a useful model for gene expression and other molecular studies.

References

References and other information relating to this product are available online at www.atcc.org.

Biosafety Level: 1

Appropriate safety procedures should always be used with this material. Laboratory safety is discussed in the current publication of the *Biosafety in Microbiological and Biomedical Laboratories* from the U.S. Department of Health and Human Services Centers for Disease Control and Prevention and National Institutes for Health.

ATCC Warranty

The viability of ATCC® products is warranted for 30 days from the date of shipment, and is valid only if the product is stored and cultured according to the information included on this product information sheet. ATCC lists the media formulation that has been found to be effective for this strain. While other, unspecified media may also produce satisfactory results, a change in media or the absence of an additive from the ATCC recommended media may affect recovery, growth and/or function of this strain. If an alternative medium formulation is used, the ATCC warranty for viability is no longer valid.

Disclaimers

This product is intended for laboratory research purposes only. It is not intended for use in humans.
While ATCC uses reasonable efforts to include accurate and up-to-date information on this product sheet, ATCC makes no warranties or representations as to its accuracy. Citations from scientific literature and patents are provided for informational purposes only. ATCC does not warrant that such information has been confirmed to be accurate.
This product is sent with the condition that you are responsible for its safe storage, handling, and use. ATCC is not liable for any damages or injuries arising from receipt and/or use of this product. While reasonable effort is made to ensure authenticity and reliability of strains on deposit, ATCC is not liable for damages arising from the misidentification or misrepresentation of cultures.
Please see the enclosed Material Transfer Agreement (MTA) for further details regarding the use of this product. The MTA is also available on our Web site at www.atcc.org

Additional information on this culture is available on the ATCC web site at www.atcc.org.

© ATCC 2015. All rights reserved. ATCC is a registered trademark of the American Type Culture Collection. [01/06]

CERTIFICATE OF ANALYSIS

**ATCC® Number:**	**CRL-3234™**
**Lot Number:**	**62613657**
**Name:**	**CA77**
**Description:**	**Medullary Thyroid Cancer**
**Species:**	**Rat (*Rattus norvegicus*)**
**Volume/Ampule:**	**Approximately 1 mL**
**Date Frozen:**	**06/06/14**
**Recovery:**	**A T-25 setup at a seeding density of 7.0 x 10^4 viable cells/cm^2 reaches approximately 50% to 60% confluence in 6 days.**<br>**A T-75 setup at a dilution of 1:15 is ready to subculture in 7 to 9 days.**
**Product Format:**	**Cells cryopreserved in the appropriate cryopreservation medium**
**Expiration Date:**	**Not applicable**
**Storage Conditions:**	**Vapor phase of liquid nitrogen**

Test / Method	Specification	Result
**Ampule passage number**	Report results	73
**Population doubling level (PDL)**	Report results	Not applicable
**Total cells/ampule**<br>(Cell count using Trypan Blue stain method)	Report results	5.4 x 10^6 total cells/ampule
**Post-freeze viability**<br>(Cell count using Trypan Blue stain method)	Report results	82.8 %
**Growth properties**<br>(Visual observation method)	Report results	Adherent
**Morphology**<br>(Visual observation method)	Report results	Epithelial-like
**Test for mycoplasma contamination**<br>Hoechst DNA stain (indirect) method<br>Agar culture (direct) method	 None detected<br>None detected	 None detected<br>None detected
**Species determination: COI assay (interspecies)**	Rat	Rat
**Sterility test (BacT/ALERT 3D)**<br>iAST bottle (aerobic) at 32°C<br>iNST bottle (anaerobic) at 32°C	 No growth<br>No growth	 No growth<br>No growth

ATCC (American Type Culture Collection)
P.O. Box 1549
Manassas, VA 20108 USA
www.atcc.org

800-638-6597 or 703-365-2700
Fax: 703-365-2750
E-mail: tech@atcc.org
or contact your local distributor

CONFIDENTIAL AND PROPRIETARY
This document contains proprietary information which may not be reproduced, transcribed, or conveyed in any way or for any purpose without the prior written consent of ATCC.
Template Doc ID: 31194 Template Revision: 3 Template Effective Date: 01/31/2013

ATCC

CERTIFICATE OF ANALYSIS

ATCC® Number: **CRL-3234™**
Lot Number: **62613657**

* Epithelial-like: Any adherent cells of a polygonal shape with clear, sharp boundaries between them.

Kim Ellis

Digitally signed by Kim Ellis
DN: cn=Kim Ellis, o=ATCC, ou=Quality Assurance, email=kellis@atcc.org, c=US
Date: 2014.11.25 13:23:11 -05'00'

Manager of Material Release; Quality Assurance

ATCC hereby represents and warrants that the material provided under this certificate is pure and has been subjected to the tests and procedures specified and that the results described, along with any other data provided in this certificate, are true and correct to the best of the company's knowledge and belief. This certificate does not extend to the growth and/or passage of any living organism or cell line beyond what is supplied within the container received from ATCC.

This product is intended to be used for laboratory research use only. It is not intended for use in humans, animals, or for diagnostics. Appropriate Biosafety Level (BSL) practices should always be used with this material. Refer to the Product Information Sheet for instructions on the correct use of this product.

ATCC products may not be resold, modified for resale, used to provide commercial services, or to manufacture commercial products without prior written agreement from ATCC.

The ATCC trademark and trade name and any and all ATCC catalog numbers are trademarks of the American Type Culture Collection.

© 2010 ATCC. All rights reserved.

ATCC (American Type Culture Collection)
P.O. Box 1549
Manassas, VA 20108 USA
www.atcc.org

800-638-6597 or 703-365-2700
Fax: 703-365-2750
E-mail: tech@atcc.org
or contact your local distributor

CONFIDENTIAL AND PROPRIETARY
This document contains proprietary information which may not be reproduced, transcribed, or conveyed in any way or for any purpose without the prior written consent of ATCC.
Template Doc ID: 31194 Template Revision: 3 Template Effective Date: 01/31/2013

ATCC

ATCC Cell Lines: Direct from the source

ATCC maintains the highest standards for its biological materials. Stringent quality control procedures produce cell lines that are well characterized and have low passage numbers. ATCC cell lines are the standard for researchers around the world.

Summit Pharmaceuticals is Japan's only authorized distributor of authentic ATCC materials. The cell lines come directly from ATCC and are not regrown or repackaged. Long-term subculturing of cell lines can have adverse effects on a cell population, including genetic drift and unwanted selection methods. The use of seed stock minimizes the number of passages and keeps the cell line as close as possible to the original.

Don't risk valuable research time on questionable materials. Use reliable ATCC cell lines, only from Summit.

For more information, contact....

Summit Pharmaceuticals International Corporation
Harumi Island Triton Square Office Tower Z
1-8-12, Harumi, Chuo-ku
Tokyo 104-6223, Japan

Attention: ATCC Group

Telephone: (81) 3-3536-8640
Fax : (81) 3-3536-8641
Email: atcc@summitpharma.co.jp
Internet: www.summitpharma.co.jp

This arrangement between ATCC and Summit is part of our commitment to providing authentic research materials and standards worldwide, and we are confident that it will benefit biological and biomedical research throughout Japan.

納 品 書

納品書No　AD1604020

お客様ID　CU025355
依頼書No　ASE1604004
オーダーNo　AD160401C002

ご納品予定日　2016年4月21日
※記載された納品予定日は交通事情等により実際の納品日とずれる場合がございますことご了承ください。

荷受主:　国立大学法人　浜松医科大学　殿
販売先:　株式会社カーク　殿
下記とおり御送付申し上げましたから貴着御査収願います。

〒104-6223
東京都中央区晴海1-8-12晴海トリトンスクエアZ棟
TEL:03-3536-8640 FAX:03-3536-8641
住商ファーマインターナショナル株式会社

責任者印	担当者印	
乙黒		筏津

	商品コード	品 名	数量	
1	CRL-3234	ATCラット細胞株<br>CA-77 (thyroid C cells, neuronal-like, medullary thyroid carcinoma, rat)	1<br>ampoule	

備考

商品受領書

AD1604020

お客様ID　CU025355
依頼書No　ASE1604004
オーダーNo　AD160401C002

ご納品予定日　2016年4月21日

荷受主:　国立大学法人　浜松医科大学　殿
販売先:　株式会社カーク　殿

〒104-6223
東京都中央区晴海1-8-12晴海トリトンスクエアZ棟
TEL:03-3536-8640 FAX:03-3536-8641
住商ファーマインターナショナル株式会社
*お手数ではございますが、当書類はFAXかご郵送で弊社宛ご返信くださいますようお願い申し上げます。

	商品コード	品名	数量	
1	CRL-3234	ATCラット細胞株<br>CA-77 (thyroid C cells, neuronal-like, medullary thyroid carcinoma, rat)	1<br>ampoule	

貴社より受け取りました上記商品は荷姿の異常及び内容品に損傷、不足等の事故はありませんでした。

2011年4月22日
ご担当者様名: 佐々木[illegible]　印

ご署名・ご捺印の上、切り離さずに本紙をそのまま弊社宛にご返送ください。　(受領書が複数ページにわたっている場合にはお手数ですが一式ご返送をお願い申し上げます。)
