# Supplementary figures and images for: G ATA2 mediates the negative regulation of the prepro-thyrotropin-releasing hormone gene by liganded T3 receptor β2 in the rat hypothalamic paraventricular nucleus
Source: PLoS One. 2020 Nov 17;15(11):e0242380. doi: 10.1371/journal.pone.0242380 (PMC7671546; doi:10.1371/journal.pone.0242380)

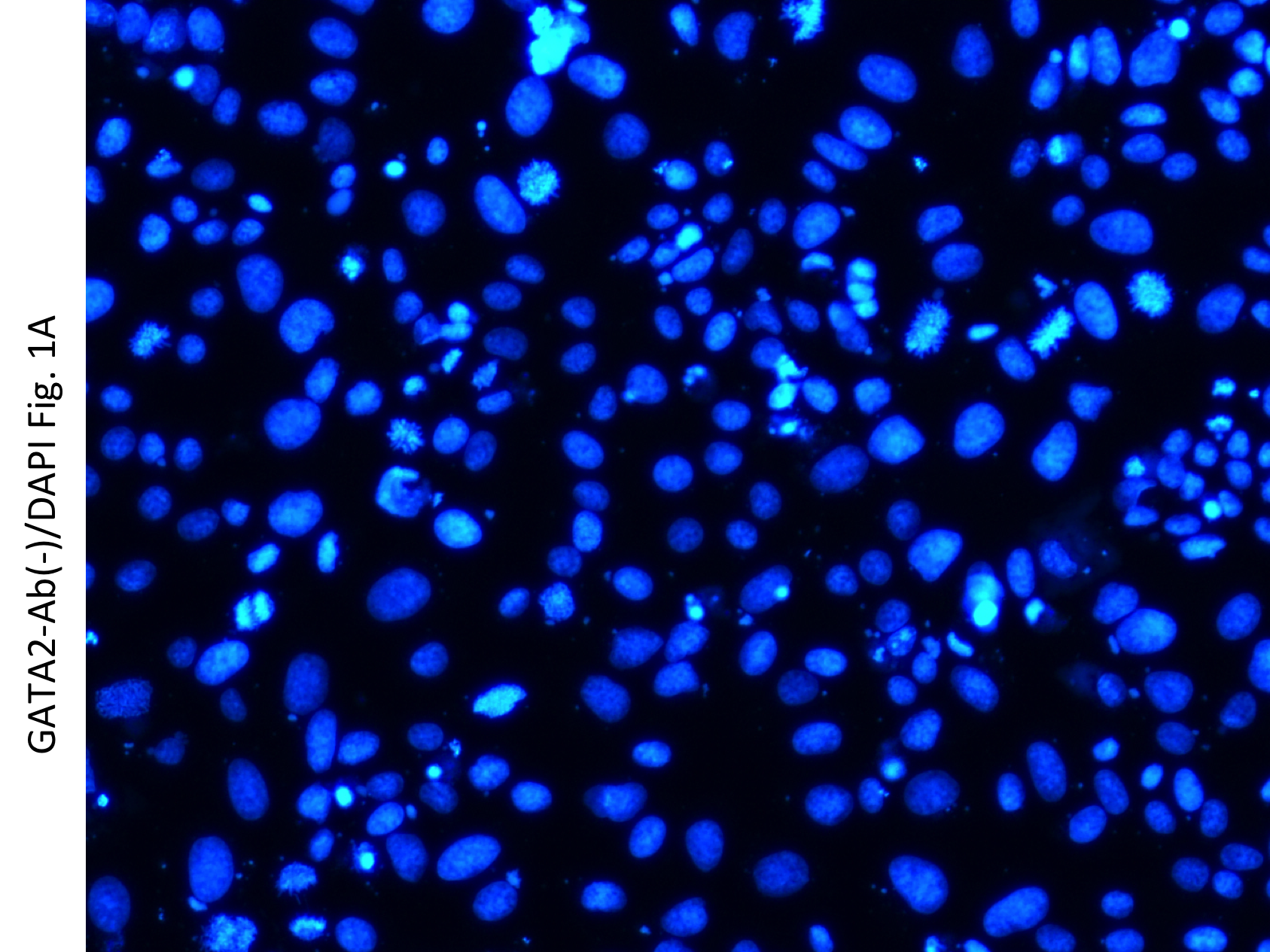

Supplement: S1 Fig — (TIF) [file pone.0242380.s003.tif]

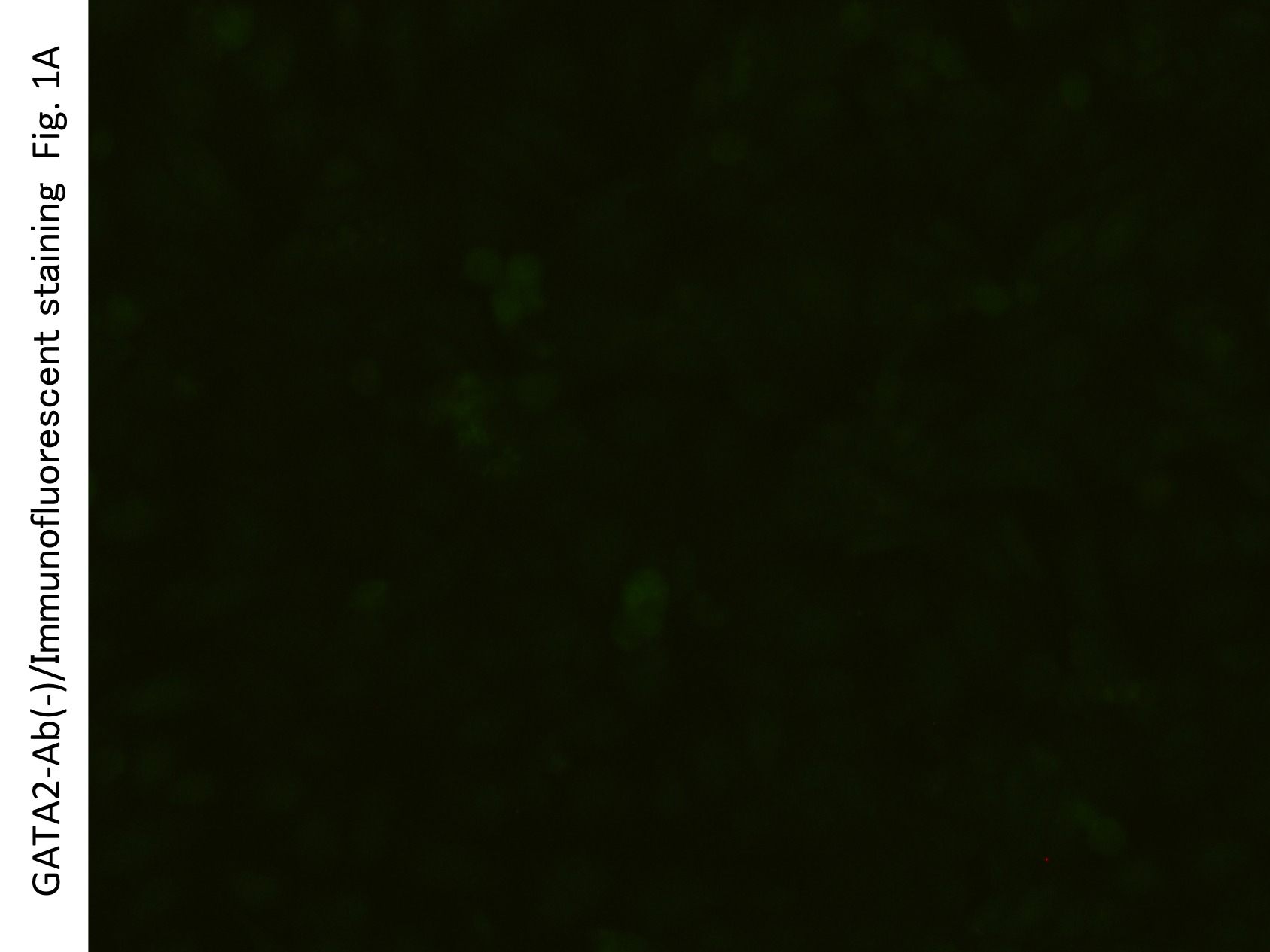

Supplement: S2 Fig — (TIF) [file pone.0242380.s004.tif]

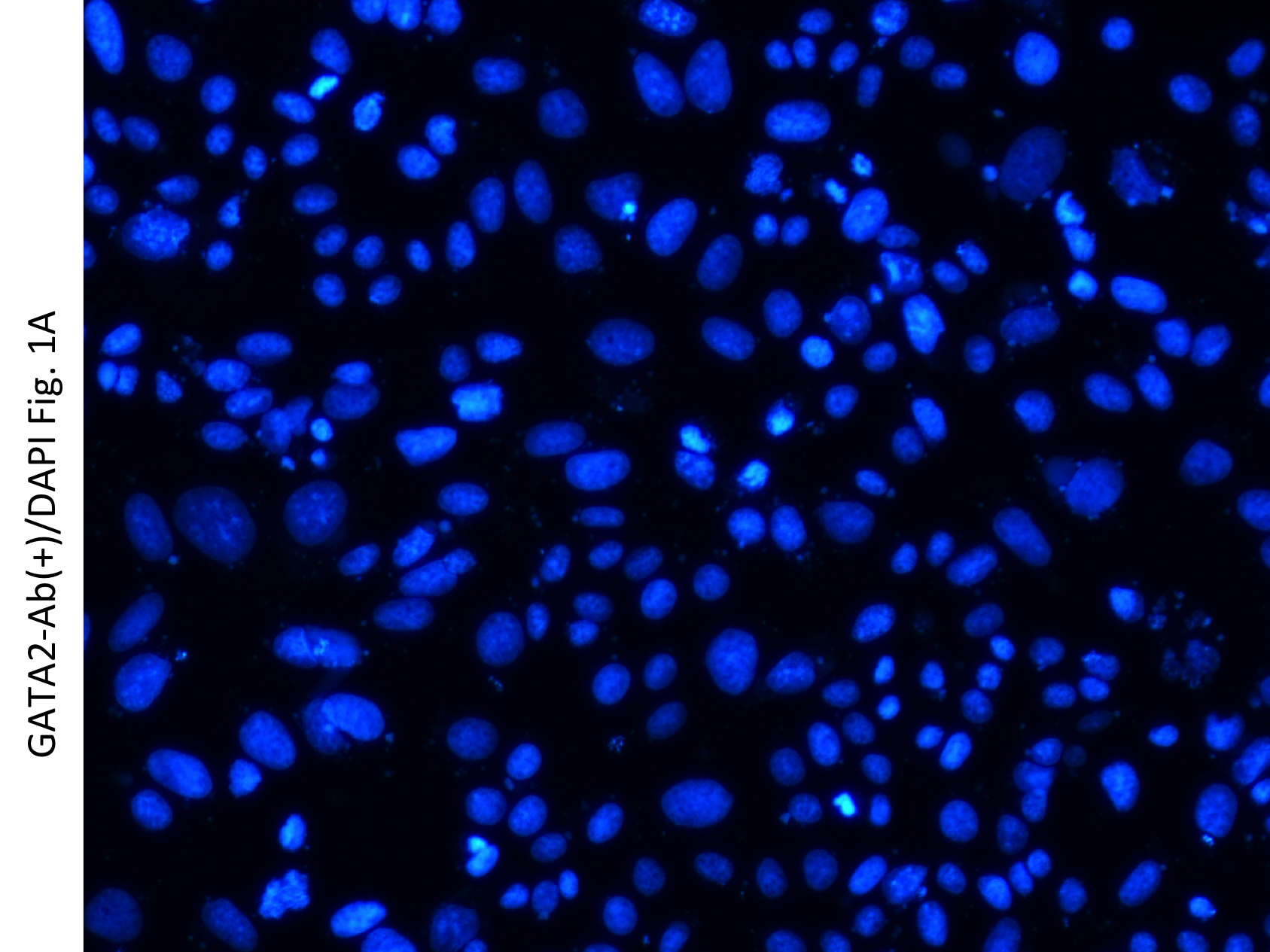

Supplement: S3 Fig — (TIF) [file pone.0242380.s005.tif]

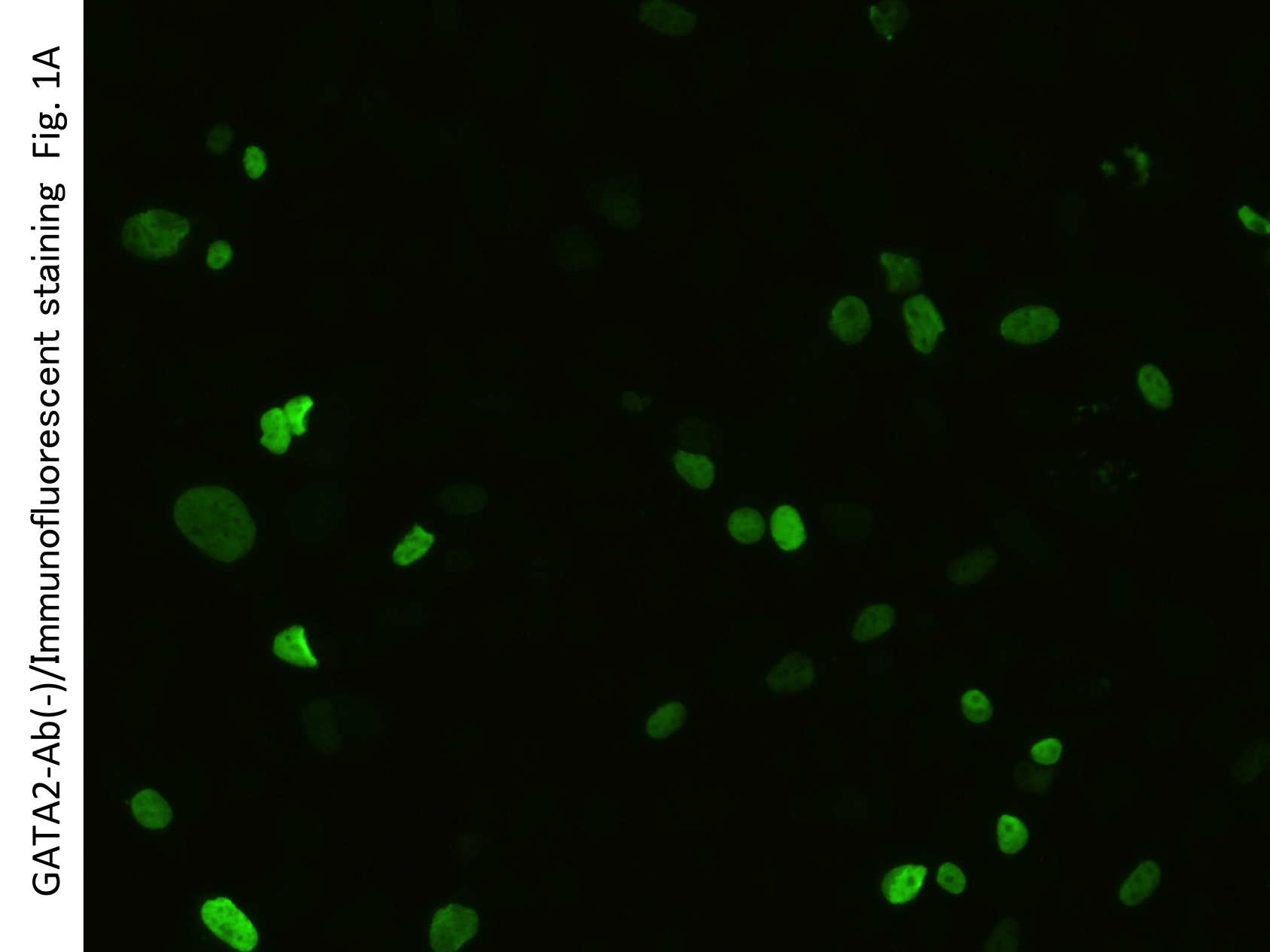

Supplement: S4 Fig — (TIF) [file pone.0242380.s006.tif]

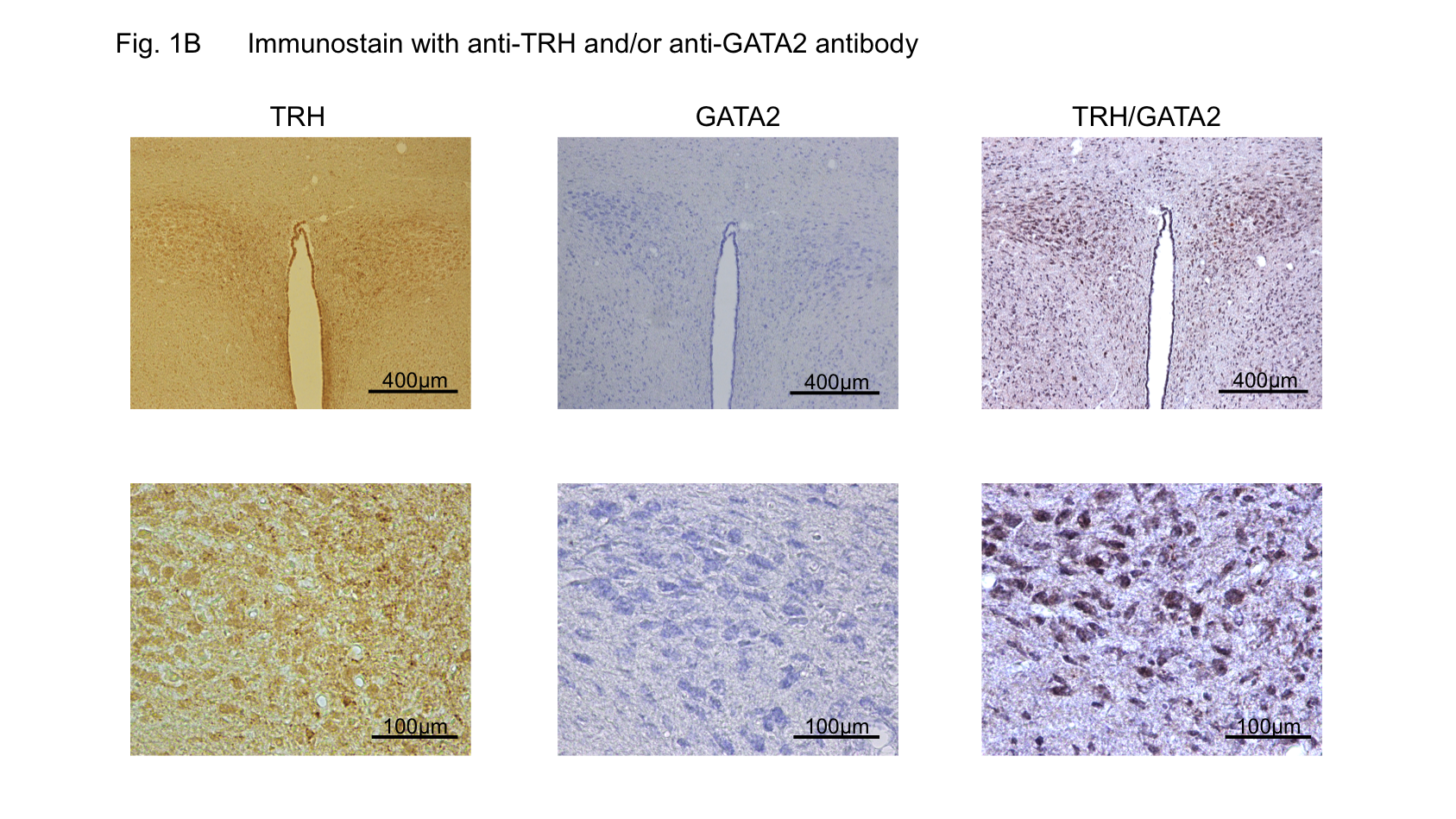

Supplement: S5 Fig — (TIF) [file pone.0242380.s007.tif]

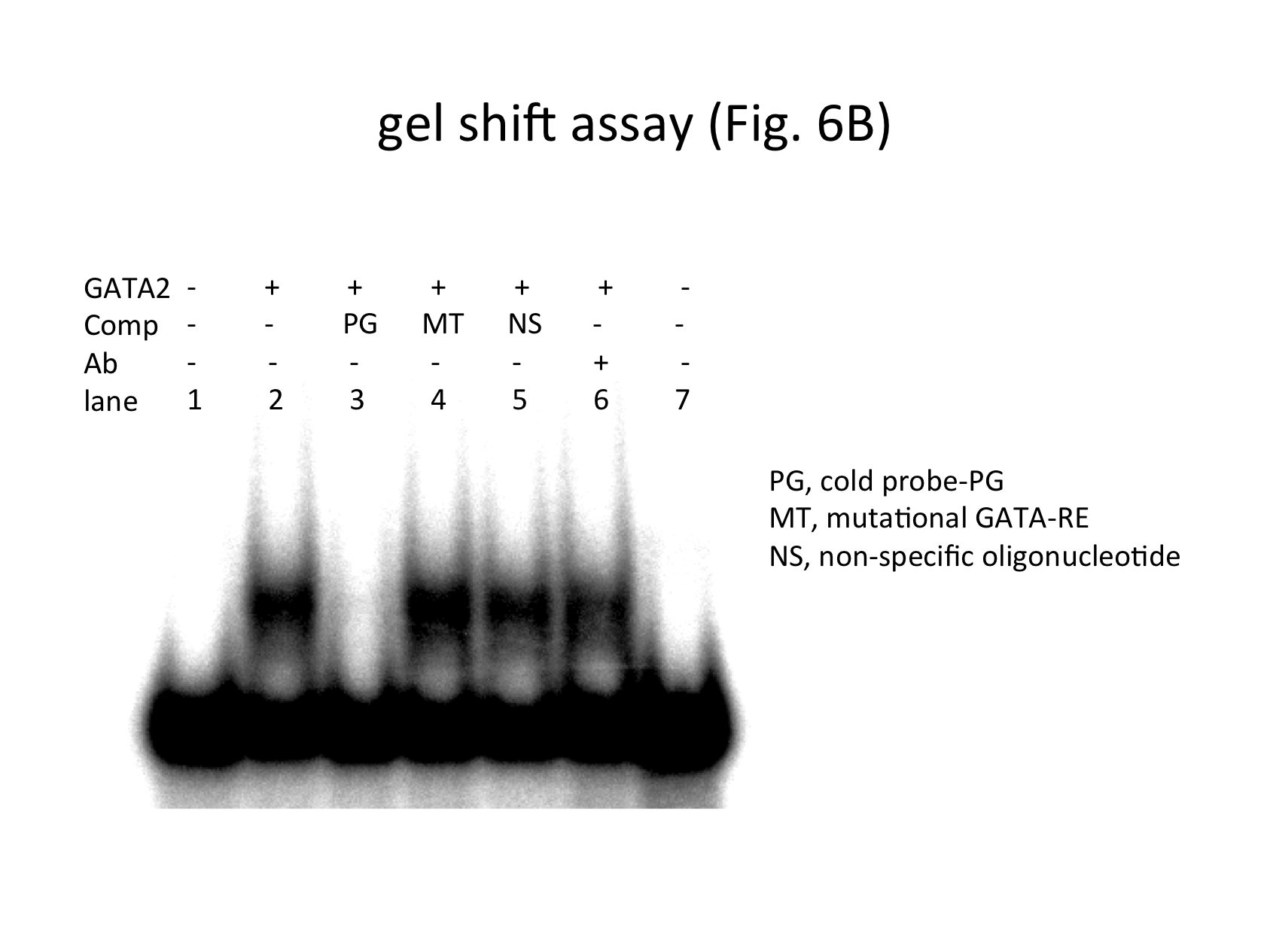

Supplement: S6 Fig — (TIF) [file pone.0242380.s008.tif]

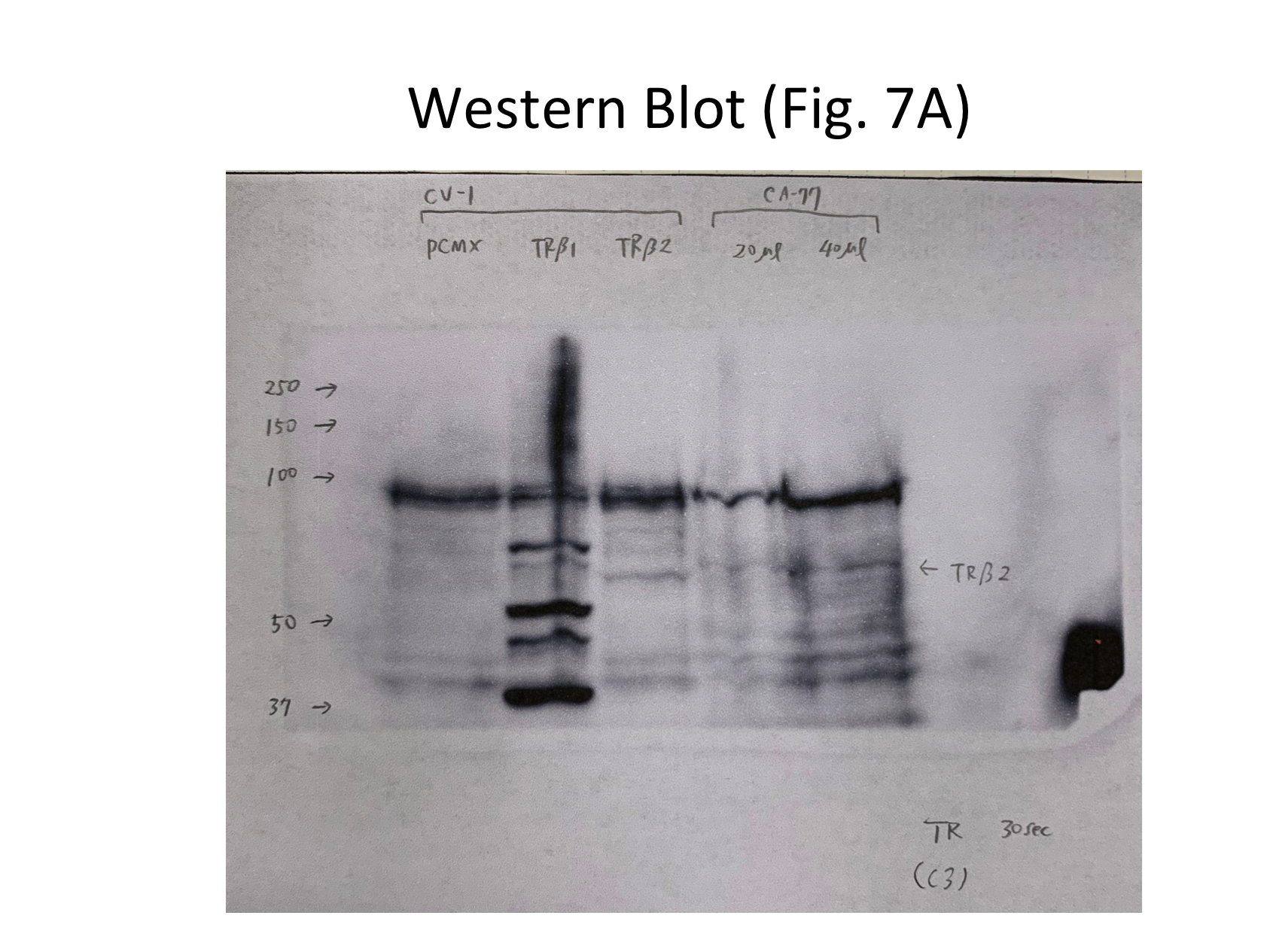

Supplement: S7 Fig — (TIF) [file pone.0242380.s009.tif]

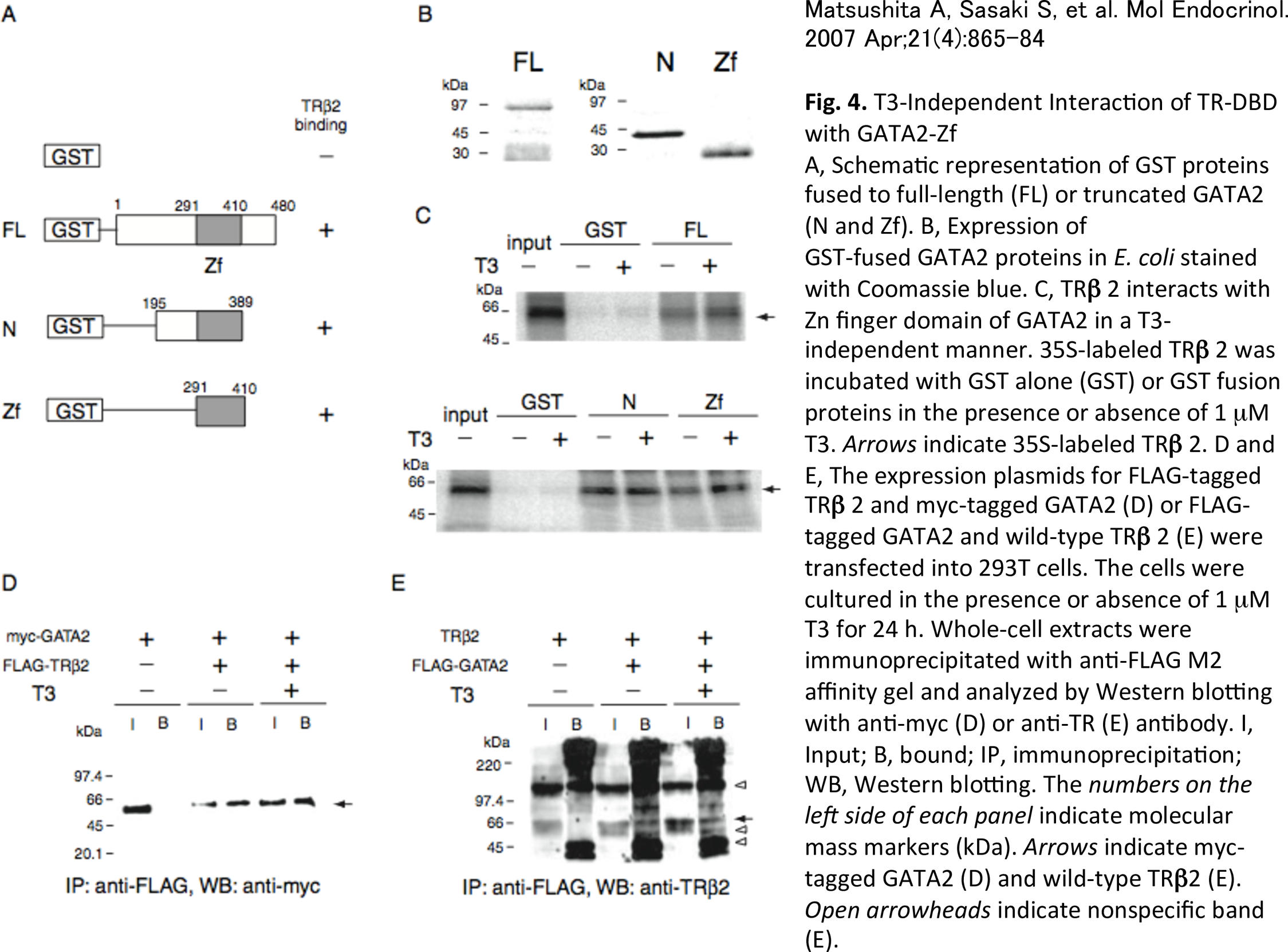

Supplement: S8 Fig — (TIF) [file pone.0242380.s010.tif]

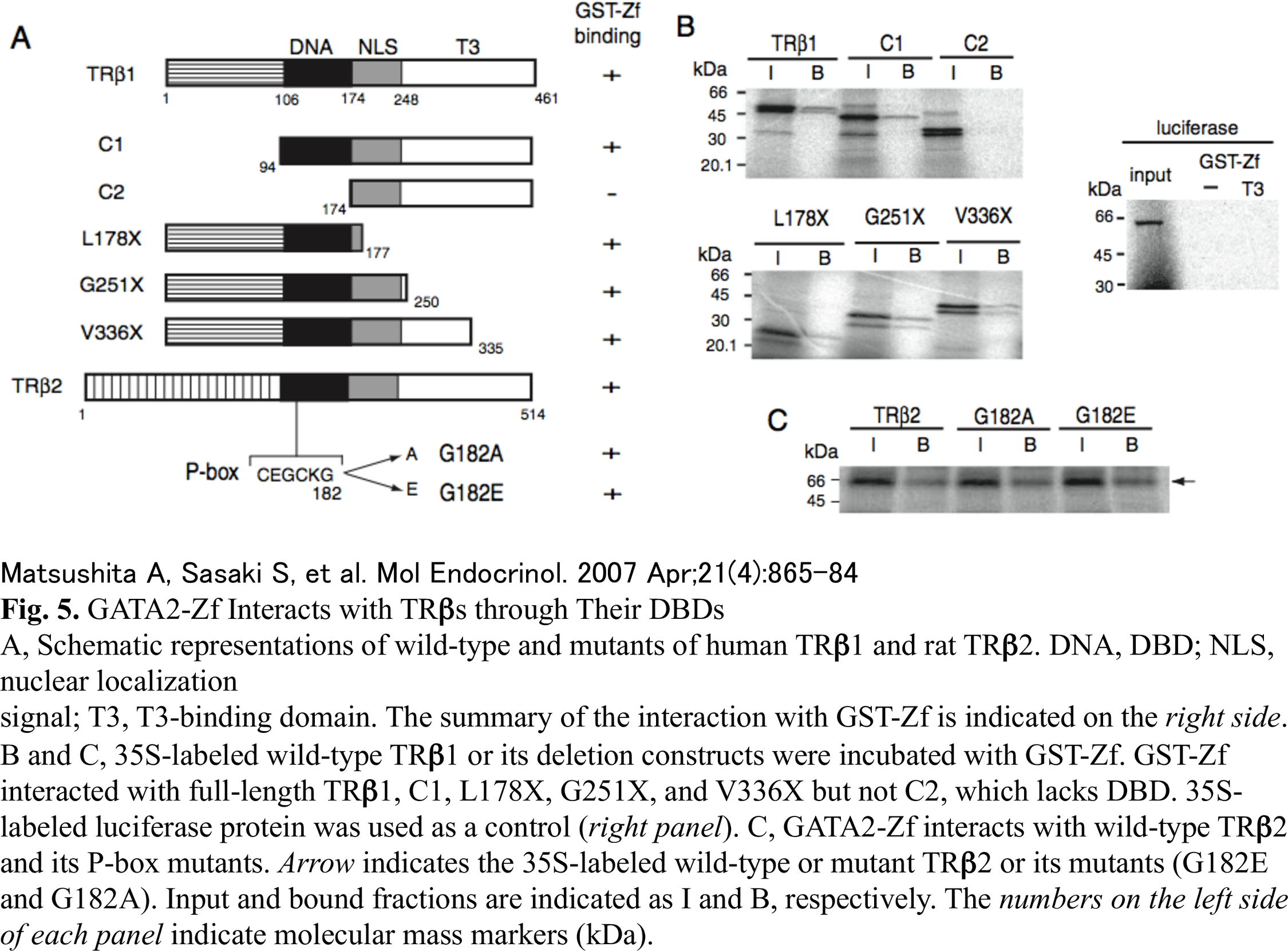

Supplement: S9 Fig — (TIF) [file pone.0242380.s011.tif]

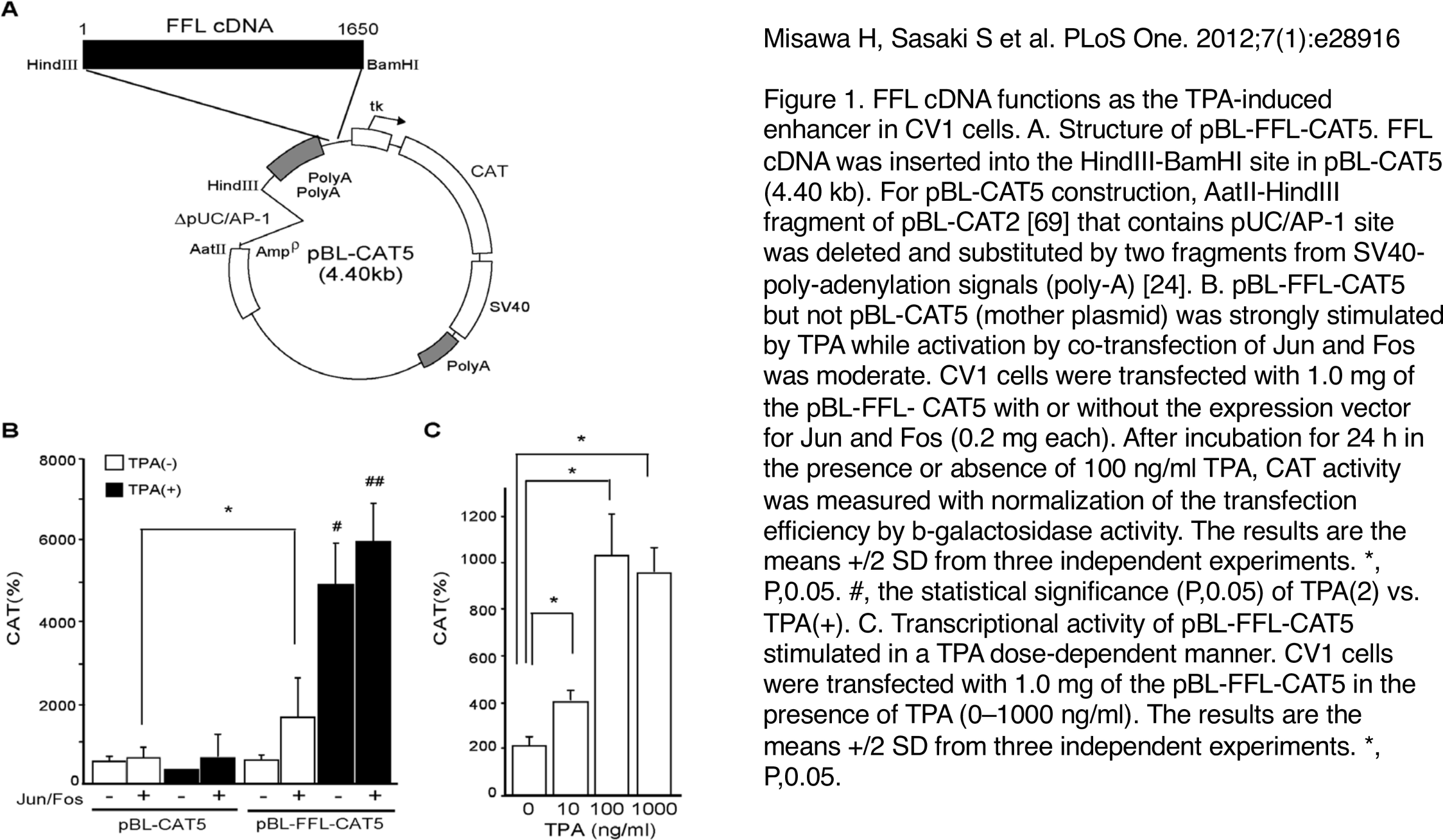

Supplement: S10 Fig — (TIF) [file pone.0242380.s012.tif]

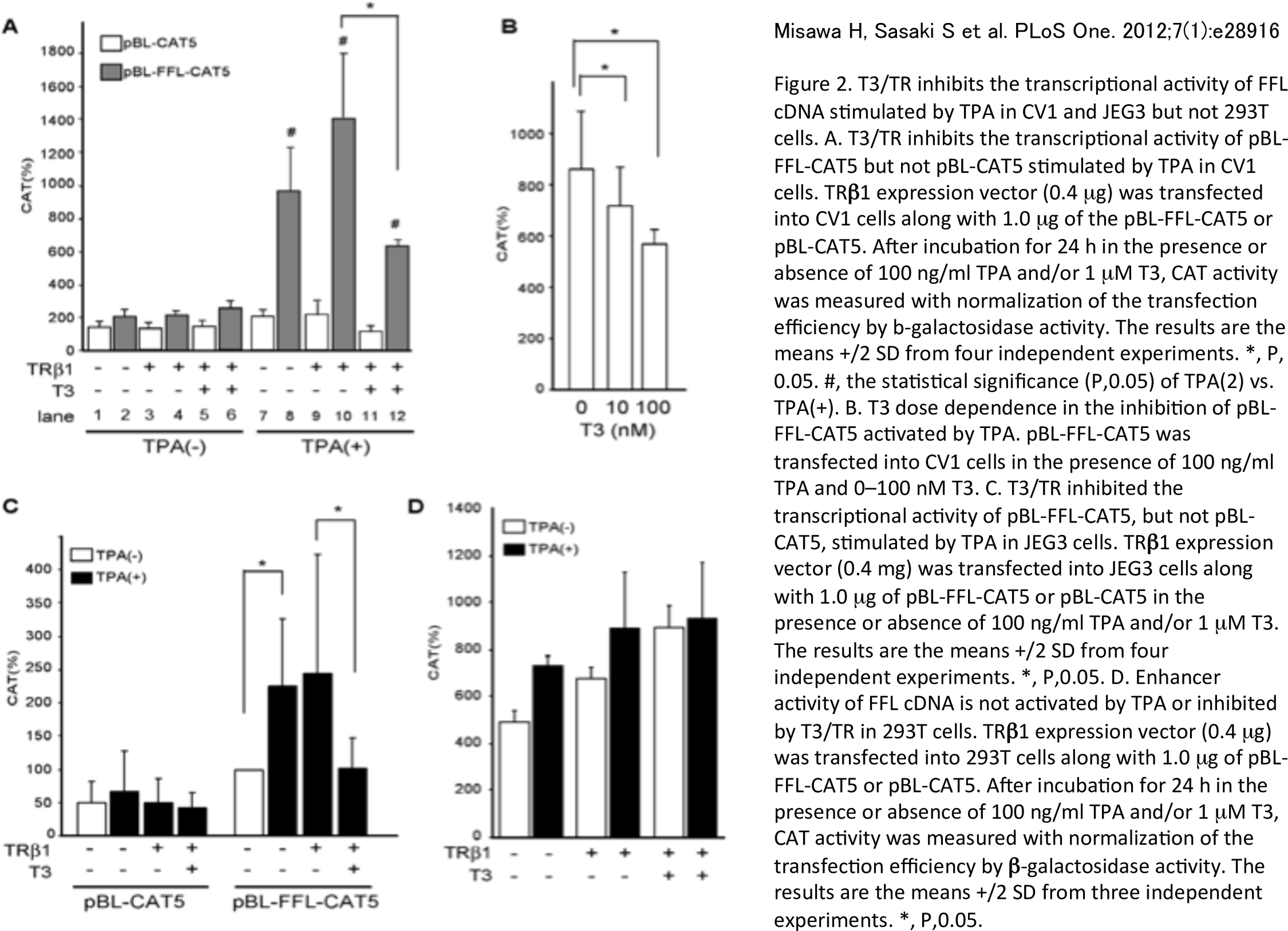

Supplement: S11 Fig — (TIF) [file pone.0242380.s013.tif]
